# Secondary Particulate Matter Originating from an Industrial Source and Its Impact on Population Health

**DOI:** 10.3390/ijerph120707667

**Published:** 2015-07-08

**Authors:** Cristina Mangia, Marco Cervino, Emilio Antonio Luca Gianicolo

**Affiliations:** 1 Institute of Atmospheric Sciences and Climate, National Research Council, s.p. Lecce-Monteroni km 1.2, 73100 Lecce, Italy; E-Mail: m.cervino@isac.cnr.it; 2 Institute of Clinical Physiology, National Research Council, s.p. Lecce-Monteroni km 1.2, 73100 Lecce, Italy; E-Mail: emilio.gianicolo@ifc.cnr.it; 3 Institute of Medical Biostatistics, Epidemiology and Informatics, Johannes Gutenberg-Universität, Mainz 55099, Germany; E-Mail: gianicolo@uni-mainz.de

**Keywords:** health and environmental impact, air pollution, fine particulate, exposure assessment, dispersion modelling, coal power plant

## Abstract

Epidemiological studies have reported adverse associations between long-term exposure to ambient particulate matter (PM_2.5_) and several health outcomes. One issue in this field is exposure assessment and, in particular, the role of secondary PM_2.5_, often neglected in environmental and health risk assessment. Thus, the aim of this work was to evaluate the long-term environmental and health impact of primary and secondary PM_2.5_ concentrations originating from a single industrial source. As a case study, we considered a coal power plant which is a large emitter of both primary PM_2.5_ and secondary PM_2.5_ precursors. PM_2.5_ concentrations were estimated using the Calpuff dispersion model. The health impact was expressed in terms of number of non-accidental deaths potentially attributable to the power plant. Results showed that the estimated secondary PM_2.5_ extended over a larger area than that related to primary PM_2.5_ with maximum concentration values of the two components well separated in space. Exposure to secondary PM_2.5_ increased significantly the estimated number of annual attributable non-accidental deaths. Our study indicates that the impact of secondary PM_2.5_ may be relevant also at local scale and ought to be considered when estimating the impact of industrial emissions on population health.

## 1. Introduction

Ambient particulate matter (PM) exposure has been associated with both short and long-term effects on mortality and morbidity for several causes [1,2,3,4,5,6,7]. PM has also been associated with adverse reproductive health outcomes like low birth weight, preterm birth and congenital anomalies [8,9,10,11,12,13]. Furthermore, the International Agency of Research on Cancer has classified outdoor air pollution as carcinogenic to humans (Group 1) and recently a meta-analysis of 18 studies has reported a Relative Risk (RR) of 1.09 for 10 µg/m^3^ of PM with aerodynamic diameters less than 2.5 µm (PM_2.5_), indicating a positive and statistical significant association between an increased risk of lung cancer and exposure to polluted air [14].

Questions arose regarding which particles characteristics in the PM mixture, such as size, number, source and toxicity, are responsible for the observed health effects and to what extent these effects are caused by particles directly emitted from combustion processes (primary PM) or by particles formed in the ambient air by the chemical reactions of gaseous precursors such as sulphur dioxide (SO_2_), nitrogen oxides (NO_x_) and ammonia (NH_3_) [15] during atmospheric transport (secondary PM). A sulphate component, in combination with others, was found to play a role as a modifying factor for the short-term association between PM_2.5_ and mortality in a 25-community US study [16]. Statistically significant short-term associations were found between primary PM_2.5_ components together with secondary sources of PM_2.5_ and all-cause and cardiovascular mortality in a five-year (2003–2007) case-crossover study in Barcelona, Spain [17]. This latter result confirmed findings of a previous study concerning a cohort of Californian teachers [18] and the strength of the association between organic carbon and sulphates with mortality, cardiopulmonary, ischemic heart and pulmonary diseases. Ostro and colleagues [18] discussed also several weaknesses so that the question of PM characteristics’ role in causing mortality and morbidity is still open.

Since the 1970s coal power plants have been studied as significant sources of precursor gases (mainly SO_2_ and NO_x_) of downwind formation of fine particles. In cloud-free and summer daylight conditions, gas-phase oxidation of SO_2_ to sulphuric acid (H_2_SO_4_) by hydroxyl radicals (OH) and subsequent condensation represents the main mechanism by which SO_2_ is removed and PM_2.5_ forms [19]. Furthermore, in cooler night-time conditions, emitted sulphur and nitrogen oxides may be converted to secondary organic aerosols in the presence of nitrate (NO_3_) radicals. Then, besides relevant emission of SO_2_, emissions of NO_x_ in the same plume and the ambient air content of OH, ozone (O_3_), ammonia (NH_3_) and volatile organic compounds (VOC) also play a role in the complex formation of inorganic (sulphates, nitrates) and organic secondary particulate matter.

Since the formation of secondary PM_2.5_ is time-dependent, with NO_x_ removal much quicker than SO_2_, two questions may arise: how large the impacted area is and how far from the source the secondary PM_2.5_ reaches its maximum ground level concentration. During and after the EXTERNE European project series [20], a major effort was made to understand on which scale (local, regional or hemispherical) atmospheric pollution caused from the thermoelectrical sector should be accounted for. Modelling the dispersion of emitted pollutants and the formation of secondary PM_2.5_ has been recognised as the key to addressing this issue. Since the first results from the project, it has become clear that the health damage and environmental impact might extend more than 500 km away from the sources. Nevertheless impacts and damages at local (<50 km) and regional (from 50 to 500 km) scales cannot be ignored, both for the emissions contribution from a single power plant [21,22,23] and from a regional thermoelectric power system [24,25].

Due to the complex non-linear gas-particle chemistry, modelling the formation of secondary PM_2.5_ from a single point source still presents significant challenges [23,26] and despite its contribution it is recognized as being relevant in respecting air quality standards, it is often disregarded in environmental and health impact assessments.

The main aim of this work is to evaluate the long-term environmental impact of both primary and secondary PM_2.5_ from a coal plant and to quantify the health impact on the population at local scale of the two components by varying some assumptions of the dispersion model.

## 2. Material and Methods

### 2.1. Source Characteristics and Area of Study

**Figure 1:**
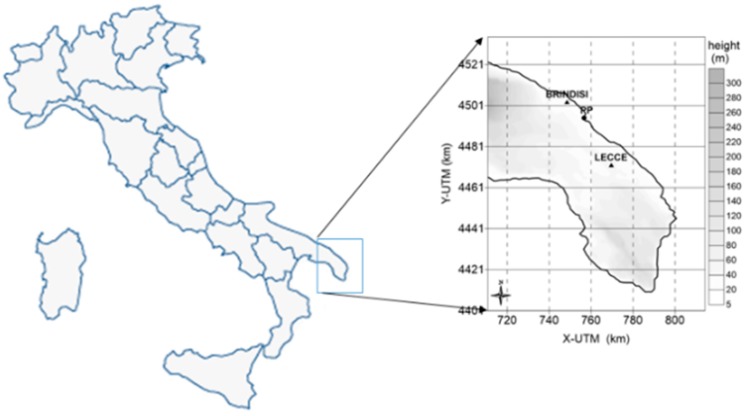
Area of study.

We considered the emissions of the coal power plant located in the municipality of Brindisi in southern Italy (Figure 1). 

With its 4 × 660 MW groups, this power plant is one of the largest in Europe. It burns about 6 × 10^6^ tonnes per year (t/y) of pulverised coal and 1–2 × 10^5^ t/y of heavy fuel oil [27]. Emissions for the year 2006 were derived from the Inventario Nazionale delle Emissioni e delle loro Sorgenti‒ European Pollutant Emission Register (INES–EPER) database [28] and are: 10,175 t/y for SO_2_, 9282 t/y for NO_x_ and 730 t/y for PM. Other basic information about the plant are the stack height (200 m), stack diameter (4 × 6.8 m), flue gas velocity (20 m/s), gas temperature (373 K) and flow rate of the flue gas (4 × 2,400,000 Nm^3^/h) [27].

The region is generally flat, with small hills (less than 200 m) in the south-eastern area and moderately high (about 500 m) in the northern part. When anticyclonic conditions affect the central Mediterranean basin, the area is dominated by a north-westerly synoptic wind (more than 50% of total wind events), intensified by the channelling effect of the Otranto Channel separating the south-eastern Italy from Albania. During weak synoptic conditions (less than 20% of total wind events), the region may be influenced by complex sea-land breeze systems caused by the diurnal heating cycle [29].

The study area 105 × 135 km^2^ comprises two towns, Brindisi (90,000 inhabitants) and Lecce (94,000 inhabitants), and 120 villages distributed over three provinces (Taranto, Brindisi and Lecce) with a total population of 1,188,311 individuals [30]. The area comprises industrial facilities such as a steel factory, two more power plants, a petrochemical plant and incinerators. 

Epidemiological studies have revealed critical situations in terms of high values for mortality and morbidity rates, consistent with environmental and occupational exposure to pollutants, for which the contributions of ports and the industrial sector have been hypothesized [31,32].

### 2.2. Atmospheric Modelling Setup

We used the Calmet/Calpuff modelling system to estimate the impact of the power plant emissions on air quality [33]. The modelling system is recommended by the Environmental Protection Agency for simulating long-range transport [34], it has been applied in previous power plant exposure studies [21,24,35,36,37] and has been already applied in the investigated area [38]. 

Calmet is a diagnostic meteorological model that generates an hourly three-dimensional meteorological field on a gridded modelling domain. 

Calpuff is a Lagrangian non-steady state puff model that allows for the handling of complex three-dimensional winds in terrain as complex as the coastal areas, it can treat calm wind conditions. It includes parametrised chemistry modules for the formation of secondary sulphate and nitrate from the oxidation of the primary gas pollutants SO_2_ and NO_x_. The chemical mechanisms considered were: MESOPUFF, a five species scheme (SO_2_, SO_4_^+^, NO*_x_*, HNO_3_, NO_3_^−^); and RIVAD/ARM3, which treats the NO and NO_2_ oxidation process in addition to the NO_2_ to NO_3_ and SO_2_ to sulphate (SO_4_) chemical transformations. MESOPUFF is the preferred scheme of the US EPA and it is generally appropriate for most applications. RIVAD/ARM3 has been stated to be more appropriate in rural areas [33]. Constant night-time gas-phase SO_2_ and NO_x_ conversion rates are specified as default values in the model. Daytime SO_2_ and NO_x_ oxidation are hourly varying functions of background O_3_ concentration, solar radiation, atmospheric stability and plume NO_x_ concentration. Two options are available for background O_3_ data: a single, typical background value appropriate for the modelling domain and O_3_ data from one or more monitoring stations.

We considered a 105 km × 135 km Calmet/Calpuff modelling domain with a resolution of 1.5 km × 1.5 km and height of 3000 m with six levels (0, 19, 100, 300, 750 and 3000). Simulations were performed for the year 2006.

Several modelling options drive the estimation of secondary PM_2.5_ formation in Calmet/Calpuff system (further details are available in Appendix A). Keeping in mind that the main goal of our work is not a complete sensitivity analysis of the model, but to underline how some different simulation choices may change the health impact estimations of secondary PM_2.5_ with respect to that caused by primary PM_2.5_ emissions, six model runs were carried out by varying the two gas-particle conversion mechanisms and key input variables such as background O_3_ and NH_3_ concentration. For background O_3_ data we assumed the Calpuff default value of 80 ppb constant in time and space and the ozone concentration data measured in two monitoring stations, whose statistics are reported in Table 1 [39]. For background NH_3_ values, we assumed the Calpuff default value of 10 ppb as in a previous work of Levy and coauthors [24]. Due to the unavailability of measured data, we selected an alternate lower value of 5 ppb, because even lower values (0.1–1 ppb) seemed appropriate for areas very far from any anthropogenic ammonia sources, but this is not the case for our area of study. Table 2 summarises the six model runs. Sensitivity on NH_3_ input were performed considering only the ozone measured data which are more reliable.

**Table 1:** Location, minimum, maximum, average and standard deviation of hourly O_3_ concentration measurements at two monitoring stations located within the simulation domain. Year 2006.

Station	Location		O_3_ Concentration			
	Xutm (km)	Yutm (km)	Min (ppb)	Max (ppb)	Average (ppb)	St. Dev (ppb)
S1	749.277	4503.418	2	79	32	14
S2	764.807	4478.158	4	115	38	15

**Table 2:** Characteristics of different Calpuff runs for the estimation of secondary particulate matter (PM_2.5_) emitted by the coal power plant located in Brindisi (Italy). Year 2006.

Run	O_3_ (ppb)	NH_3_ (ppb)	Chemical Mechanism
A1	80	10	MESOPUFF
B1	Monitored data	10	MESOPUFF
C1	Monitored data	5	MESOPUFF
A2	80	10	RIVAD/ARM3
B2	Monitored data	10	RIVAD/ARM3
C2	Monitored data	5	RIVAD/ARM3

### 2.3. Health Impact

Up to now, no cohort study has been conducted in this area to estimate the long-term effects of air pollution exposure. Several meta-analyses were published on the association between exposure to ambient PM_2.5_ and non-accidental mortality [40,41,42]. However, in this work, in order to estimate the long-term impact of primary and secondary PM_2.5_ on mortality for non-accidental causes, a hazard ratio (HR) estimation of 1.07 (95% CI 1.02–1.13) for a 5 μg/m^3^ increment of PM_2.5_ was chosen based on a recent and large European multicenter study [7]. To estimate the number of non-accidental deaths potentially attributable to increased PM_2.5_ levels, we derived a three-step procedure [43].

First, we computed the baseline population frequency (P_0i_), *i.e.*, the proportion of the population, for the *i*th municipality, that would experience the outcome assuming a null air pollution level:

(1) $$P_{0i}≅\frac{P_{ei}}{\left[1+\frac{\left(RR-1\right)}{5}\cdot E_{pm}\right]}$$

where *P_ei_* is the observed annual mortality in the area and *RR* is the *HR* observed by [7] for a 5 μg/m^3^ increase in PM_2.5_; therefore division by 5 was done. *E_pm_* is the observed population average PM_2.5_ exposure level, here assumed equal to 20 μg/m^3^, which is the rounded annual (2011) average value measured in the area [44].

The fixed baseline increment, *D*_1*i*_, of deaths per a reference population (e.g., 100,000) is calculated assuming a linear additive effect of air pollution above the zero level as the delta for a 1 µg/m^3^ increment in PM_2.5_.

(2) $$D_{1_{i}}=100,000\cdot P_{0i}\cdot \frac{\left(RR-1\right)}{5}$$

To estimate a range of impact rather than a point estimate, the upper and lower 95% confidence interval values of the *RR* were used in the previous equation. Lower and upper *P_0i_* and *D*_1i_ values could then be derived.

Finally, the estimated number of additive attributable cases over the domain area is computed by estimating the annual average of PM_2.5_ concentration in air attributable to the power plant in each of the municipalities, together with their population, by summing:

(3) $$AC=\sum_{i=1}^{120}\frac{D_{1i}\cdot P_{i}}{100,000}\cdot X_{i}=\frac{\left(RR-1\right)/5}{\left[1+\frac{\left(RR-1\right)}{5}\cdot E_{pm}\right]}\cdot \sum_{i=1}^{120}N_{i}\cdot X_{i}$$

where *AC* is the attributable additional deaths, *P_i_* is the municipality’s population, *N_i_* is the municipality’s number of deaths for natural causes and X*_i_* is the annual average of PM_2.5_ concentration in the air attributable to the power plant.

For each municipality in the study area, we used the resident population and the number of total deaths (11,571) reported by the National Institute of Statistics for 2006 [30,45]. Since it was not possible to obtain mortality for natural-cause at a municipal level, this was estimated by assuming a percentage of accidental causes equal to that observed at the provincial level.

## 3. Results

**Figure 2:**
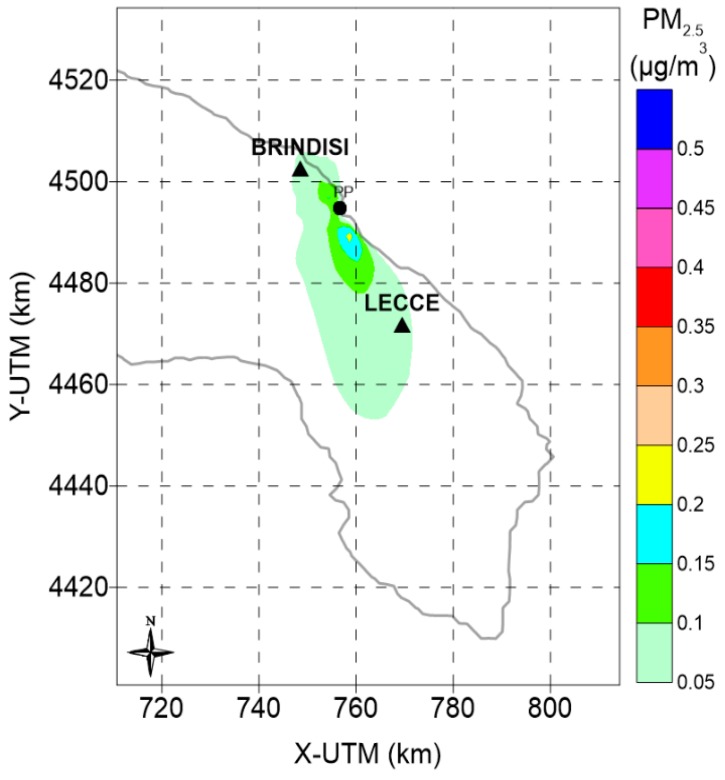
Estimated annual average primary PM_2.5_ concentrations (µg/m^3^). Year 2006.

Figure 2 shows the modelled annual average concentration of primary PM_2.5_.

**Figure 3:**
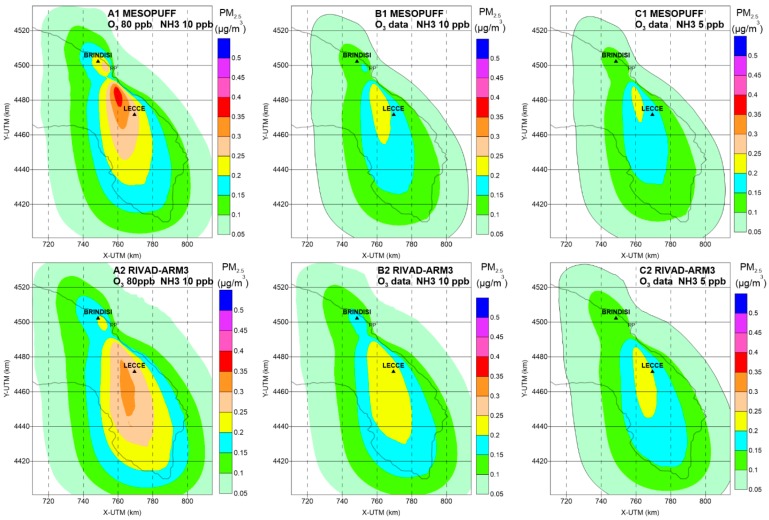
Estimated annual average secondary PM_2.5_ concentrations (µg/m^3^) for different Calpuff runs. Year 2006.

**Figure 4:**
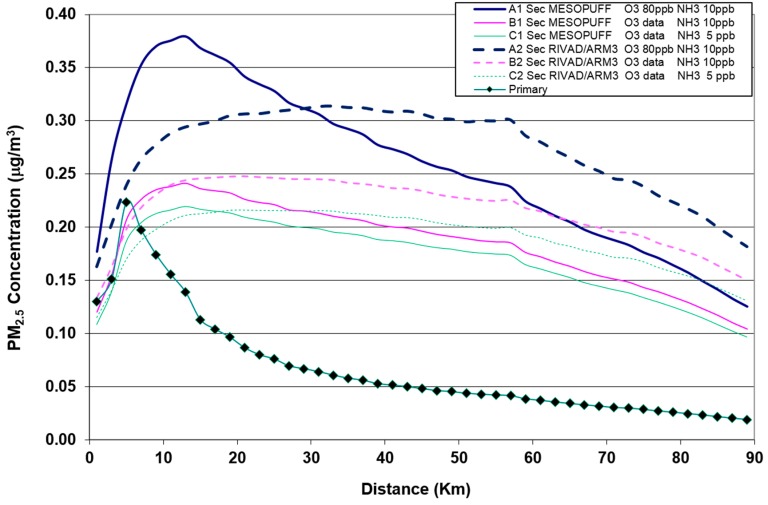
Estimated maximum annual average PM_2.5_ concentrations (µg/m^3^) plotted against distance from the source for different Calpuff runs.

Figure 3 depicts the patterns and magnitudes of secondary inorganic PM_2.5_ obtained by varying O_3_ and NH_3_ background concentration values and the chemical mechanism (Table 2). Figure 4 shows the maximum concentrations as a function of the distance from the source. 

By comparing the primary PM_2.5_ concentration pattern with the overall secondary PM_2.5_ runs, the latter peak further from the source in a range between 12 and 32 km depending on key parameters choices and diminish more slowly with the distance from the source. It is also evident how the estimated secondary PM_2.5_ extended over a larger area with a greater spatial average between 0.07 to 0.12 μg/m^3^ (Table 3).

Varying background O_3_ from 80 ppb to measured concentration data (Run A1–B1; A2–B2) decreased secondary PM_2.5_ maximum and average values for both chemical mechanisms. Separate computations for sulphates and nitrates (not shown here) attributed this decrease to a computed lower sulphate production. Decreasing background NH_3_ from 10 ppb to 5 ppb (B1–C1, B2–C2) also led to a decrease of secondary PM_2.5_, but in this case the decrease was brought by the nitrate component. Moreover, RIVAD/ARM3 produces maximum concentrations at a greater distance and a higher spatial average concentration with respect to estimates obtained with MESOPUFF chemical mechanism.

**Table 3:** Minimum, maximum, average over the simulation domain PM_2.5_ concentration for the primary PM_2.5_ emission and six runs for secondary PM_2.5_ formation, along with the distance from the source of the location of the maximum value. Year 2006.

Run	Minimum (μg/m^3^)	Maximum (μg/m^3^)	Average (μg/m^3^)	Distance of the maximum from the source (Km)
Primary	0.00	0.22	0.02	6
A1	0.01	0.38	0.10	12
B1	0.01	0.24	0.08	12
C1	0.01	0.22	0.07	12
A2	0.01	0.31	0.12	32
B2	0.01	0.25	0.10	22
C2	0.01	0.22	0.08	22

**Table 4:** Estimated number of non-accidental deaths and 95% confidence interval (95% CI) associated with different scenario of modelled exposure to primary and secondary particulate matter (PM_2.5_) emitted by the coal power plant located in Brindisi (Italy). Year 2006.

Scenario	Absolute Number of Cases	95% CI		Number of Cases per 100,000 Inhabitants	95% CI	
		Lower	Upper		Lower	Upper
Primary PM_2.5_	4	1	7	0.4	0.1	0.6
Secondary and primary PM_2.5_—run A1	26	9	41	2.2	0.7	3.4
Secondary and primary PM_2.5_—run B1	20	7	31	1.7	0.6	2.6
Secondary and primary PM_2.5_—run C1	19	6	30	1.6	0.5	2.5
Secondary and primary PM_2.5_—run A2	28	10	44	2.4	0.8	3.7
Secondary and primary PM_2.5_—run B2	23	8	37	2.0	0.7	3.1
Secondary and primary PM_2.5_—run C2	21	7	33	1.8	0.6	2.8

The estimated number of non-accidental deaths annually attributable to primary PM_2.5_ was 4 inhabitants (IC95% 1–7) and increased, depending on different assumptions, to 19 (IC95% 6–30) and to 28 (IC95% 10–44) when the secondary PM_2.5_ was also considered (Table 4).

## 4. Discussion

Our study showed that estimates of secondary PM_2.5_ originating from a facility with high emissions of SO_2_ and NO_X_ extend over a larger area than those related to primary PM_2.5_, with peak concentrations of the two components well separated in space. On the other hand, secondary PM_2.5_ concentration values are still around half of the peak values even near the boundary of the simulation area in the direction of the prevailing winds. This is in line with similar studies [21,22,23] investigating the impact of a single source over a comparable extension area.

The expected number of non-accidental deaths associated with the estimated exposure to primary PM_2.5_ was 4 in the entire area and increased to 19–28 events, when the secondary PM_2.5_ was also taken into account.

Some limitations must be considered when interpreting the results of this study. These regard both the dispersion model and the analysis of mortality effects. First of all, gas-particle conversion is a non-linear chemical process dominated by emitted and background values of several species but the Calpuff model uses a simplified linear mechanism with respect to gaseous precursors. Moreover, as the background concentration values of NH_3_ and O_3_ are key input parameters of the plume chemistry, uncertainties arise from the model assumption of constant values both in time and in the three-dimensional spatial domain. Different sensitivity analysis showed the manner in which the estimated exposure to secondary PM_2.5_ depends on the assumption of the internal chemical mechanism as well as on the background inputs assumed for O_3_ and NH_3_. The average and maximum values of secondary PM_2.5_ ranged from 0.07 to 0.12 µg/m^3^ and from 0.22 to 0.38 µg/m^3^, respectively. Higher sensitivity was found for chemical mechanism and O_3_ input. The use of measured O_3_ data in place of the default Calpuff value of 80 ppb decreases secondary particulate. Similar results were found by Lopez and coauthors [21]. Lower sensitivity resulted by halving (from 10 to 5 ppb) the NH_3_ background value, with resulting secondary PM_2.5_ decreasing again. Even lower NH_3_ values seemed not appropriate due to surveying modelling results obtained for rural areas in the largest Italian valley (Po Valley) that have similar land use of the area under examination [46]. These limitations could be partially overcome by using more complex grid photochemical models. Nevertheless, detailed emission inventories in the area together with initial and boundary conditions [47] are needed but often not available in environmental and health impact assessments. However, simplified models are shown to provide an acceptable screening estimate [22,23]. 

Another limitation of the study is the relative small modelling area considered, 105 × 135 km^2^. Other studies [24,37] have shown that impacts from power plant emissions can extend over 300 km from the source. Thus, an underestimation of attributable deaths might have been occurred. Although exposure to PM_2.5_ has been shown to have adverse impacts on morbidity outcomes and reproductive health, we had to limit our analysis to mortality due to the data available. Furthermore, in order to estimate the mortality for non-accidental causes, we discounted the total mortality available at a municipal level using the percentage of accidental causes available at a provincial level. In the three provinces, these percentages ranged from 4.1% to 4.9% [45].

As already pointed out in other studies [21], due to differences in population characteristics, such as socio-economic status, sex and age distribution, uncertainty exists regarding the use of risk estimates generated in settings other than those under study. Therefore, whether or not the adopted risk estimate reasonably represents the investigated settings is unclear and has to be further researched. Even the confidence interval of the relative risk considered in our analysis might indeed not be suitable in our setting. The adopted risk estimate used is based on 22 European cohort studies involving a number of participants (367,251), which is one third of the residents in Brindisi-Lecce and Taranto provinces; we could have therefore overestimated the width of the confidence interval.

In our analysis we assumed a linear relationship between PM_2.5_ exposure and mortality. This could limit our findings, although alternatives to a linear exposure-response model have been considered in other studies [5,48] and no evidence for an absence of linearity was detected.

## 5. Conclusions

This study showed that the inclusion of secondary PM_2.5_ might significantly vary the environmental and health impact estimate of a coal power plant, large emitting source of SO_2_ and NO_x_. Although the predicted annual average values seem to be rather low (less than 1 µg/m^3^), exposure to primary and secondary PM_2.5_ are expected to be associated respectively from 4 to a maximum of 28 annual non-accidental deaths. These figures ought to be considered in health protection policies. Furthermore, secondary particulate formation seems to be one of the pollution pressure that should not be ignored in environmental and health impact assessments.

## Appendix A

### Modelling System Options

Meteorological data input for Calmet were obtained by the prognostic meteorological model MM5, which ran on hour basis and on a 4 × 4 km grid size. Domain size and grid resolution were chosen to optimise the reproduction of sea breeze circulations and computing time.

Emission rates were computed by assuming a constant power load over the year and PM was assumed entirely in the PM_2.5_ range. In modelling setup we use the default settings for Calpuff whereas measured data were not available. We assumed that secondary particles were entirely in the PM_2.5_ range, as SO_4_ and NO_3_ are predominantly observed in the fine mode and used the Calpuff default geometric mass mean diameter of 0.48 μm and geometric standard deviation of 2. We assumed a nighttime oxidation rates of 0.2 and 2.0 percent h^−1^ for SO_2_ and NOx, respectively. We modeled gas phase dry deposition (for SO_2_, NO*_x_* and HNO_3_) and particle phase dry deposition (for SO_4_, NO_3_ and primary fine particles) using the model option for full treatment of spatially and temporally varying gas/particle deposition rates predicted by a resistance deposition model. As input values to the resistance deposition model, we used the model default solubility, reactivity and diffusivity for gases (SO_2_, NO*_x_* and HNO_3_) and model default size distribution for particles (SO_4_, NO_3_ and primary fine particles). For wet deposition, Calpuff uses an empirically based scavenging coefficient method. We used Calpuff default scavenging coefficients for primary PM_2.5_, SO_2_, SO_4_ and NO_3_, with liquid precipitation coefficients of 1 × 10^−4^, 3 × 10^−5^, 1 × 10^−4^ and 1 × 10^−4^ s^−1^, respectively [33]. The RIVAD/ARM3 chemistry scheme treats the NO and NO_2_ oxidation process in addition to the NO_2_ to NO_3_ and SO_2_ to SO_4_ chemical transformations, with equilibrium between gaseous HNO_3_ and particulate NH_4_NO_3_. The chemical transformation scheme requires both NO and NO_2_ emissions rates. Typically, only NOx emission rate is known, and this is expressed in terms of NO_2_ mass equivalent. We assumed NO/NOx equal to 0.9.

Total secondary PM_2.5_ were calculated summing secondary sulphate (defined as the mass of ammonium sulphate ((NH_4_)_2_SO_4_) and secondary nitrate (defined as the mass of ammonium nitrate (NH_4_NO_3_).
